# Cytosolic DNA sensing through cGAS and STING is inactivated by gene mutations in pangolins

**DOI:** 10.1007/s10495-020-01614-4

**Published:** 2020-06-12

**Authors:** Heinz Fischer, Erwin Tschachler, Leopold Eckhart

**Affiliations:** 1 Division of Cell and Developmental Biology, Center for Anatomy and Cell Biology, Medical University of Vienna, Vienna, Austria; 2 Department of Dermatology, Medical University of Vienna, Vienna, Austria

**Keywords:** DNA sensor, Innate immunity, Inflammation, Pangolin, Zoonosis, Gene loss

## Abstract

The release of DNA into the cytoplasm upon damage to the nucleus or during viral infection triggers an interferon-mediated defense response, inflammation and cell death. In human cells cytoplasmic DNA is sensed by cyclic GMP-AMP Synthase (cGAS) and Absent In Melanoma 2 (AIM2). Here, we report the identification of a “natural knockout” model of cGAS. Comparative genomics of phylogenetically diverse mammalian species showed that cGAS and its interaction partner Stimulator of Interferon Genes (STING) have been inactivated by mutations in the Malayan pangolin whereas other mammals retained intact copies of these genes. The coding sequences of *CGAS* and *STING1* are also disrupted by premature stop codons and frame-shift mutations in Chinese and tree pangolins, suggesting that expression of these genes was lost in a common ancestor of all pangolins that lived more than 20 million years ago. *AIM2* is retained in a functional form in pangolins whereas it is inactivated by mutations in carnivorans, the phylogenetic sister group of pangolins. The deficiency of cGAS and STING points to the existence of alternative mechanisms of controlling cytoplasmic DNA-associated cell damage and viral infections in pangolins.

**Electronic supplementary material:**

The online version of this article (10.1007/s10495-020-01614-4) contains supplementary material, which is available to authorized users.

## Introduction

The presence of DNA in the cytoplasm is a sign of infection with DNA viruses or damage to the nucleus or mitochondria in which endogenous DNA is normally contained [1, 2]. During mitosis the nuclear membrane disintegrates but DNA is compacted and tightly covered by histones so that it is not directly accessible to cytoplasmic proteins [3]. Cellular response systems have evolved to detect cytoplasmic DNA and to initiate processes aimed at the re-establishment of homeostasis at the tissue level. These responses include the production of type I and type III interferons, interleukin (IL)-1 and 18 which transmit a danger signal to neighboring cells and activate the immune system. At the cellular level, mislocalized DNA can trigger senescence and programmed cell death [4–6]. Cell death is particularly important in host defense against DNA viruses, such as vaccinia virus, enterovirus A71, and herpes viruses, and bacteria, such as *Mycobacterium tuberculosis* variant *bovis*, *Listeria monocytogenes*, *Legionella pneumophila*, and *Francisella tularenis*, but also in sterile inflammation and cancer [5, 6].

Cytoplasmic DNA is sensed by Absent in melanoma 2 (AIM2) and cyclic GMP-AMP synthase (cGAS). AIM2 activates inflammasome-dependent IL-1β and a pro-inflammatory mode of cell death known as pyroptosis [7–10]. cGAS binds DNA and catalyzes the production of 2ʹ3ʹ-cGAMP which is the ligand of Stimulator of interferon genes (STING) [11–13]. Subsequently, STING translocates to the Golgi and undergoes phosphorylation by TANK-binding kinase 1 (TBK1). Interferon regulatory factor (IRF) 3 is recruited and phosphorylated and the expression of interferon genes is induced [4, 14]. By an as-yet unclear mechanism, also NF-κB is activated by STING, leading to the expression of inflammatory cytokines such as tumor necrosis factor (TNF). Via TNF and other pathways, activation of the cGAS-STING pathway triggers various types of programmed cell death including, necroptosis, apoptosis, and lysosomal cell death [5, 6].

Cytoplasmic DNA sensors have originated early in evolution [15] and homologs of AIM2 and cGAS were present in the first mammals [16–18]. Surprisingly, AIM2 is not conserved in cattle, dog, bats, and several other mammals [18–20], suggesting inter-species differences in the response to cytoplasmic DNA and dispensability of AIM2-mediated DNA-sensing for some species.

Pangolins are specialized insectivorous mammals that are phylogenetically most closely related to carnivorans. The body of pangolins is covered by keratinous scales which serve as a protective armor. Few comparative studies of the mammalian immune defense have included pangolins but the interest in pangolins has increased recently due their possible role as intermediate hosts for the pandemic severe acute respiratory syndrome coronavirus 2 (SARS-CoV-2) [21–23]. We have recently reported that interferon-induced with helicase C domain 1 (IFIH1)/MDA5, a sensor of intracellular double-stranded RNA, and Z-DNA-binding protein (ZBP1), which senses both Z-RNA and Z-DNA, have been lost during the evolution of pangolins [24]. Moreover, toll-like receptor (TLR) 5, the receptor of bacterial flagellin [25] and interferon-ε, a type I interferon that is expressed in epithelia of other mammals, have been lost in pangolins [26].

Here we investigated whether the genes controlling cytoplasmic DNA-sensing are conserved in pangolins and found that pseudogenization of critical genes has inactivated the cGAS-STING pathway that is implicated in the innate defense against DNA viruses and cytoplasmic DNA-stimulated cell death.

## Materials and methods

Genes were identified in the genome sequences of the Malayan pangolin (*Manis javanica*), Assembly: ManJav1.0 (GCA_001685135.1), submitted by the International Pangolin Research Consortium (Choo et al. [26]); Chinese pangolin (*Manis pentadactyla*), Assembly: M_pentadactyla-1.1.1 (GCA_000738955.1), submitted by Washington University; Tree pangolin (*Manis tricuspis*), Assembly: ManTri_v1_BIUU (GCA_004765945.1), submitted by Broad Institute. At the time of this study (April 2020) GenBank gene annotations were available for *M. javanica* (NCBI Manis javanica Annotation Release 100) but not for the other species of pangolins. Other nucleotide sequences were downloaded from GenBank and accession numbers are indicated in the text.

The Basic Local Alignment Search Tool (BLAST) was used to identify regions of sequence similarity [27]. Nucleotide sequence were translated into amino acid sequences using the Translate tool at the Expasy website of the Swiss Institute of Bioinformatics (https://web.expasy.org/translate/). Sequence alignments were made with Multalin [28]. The Timetree website was used as a reference for phylogenetic relationships and divergence times (www.timetree.org) [29].

## Results

### cGAS is inactivated by gene mutations in pangolins

**Fig. 1:**
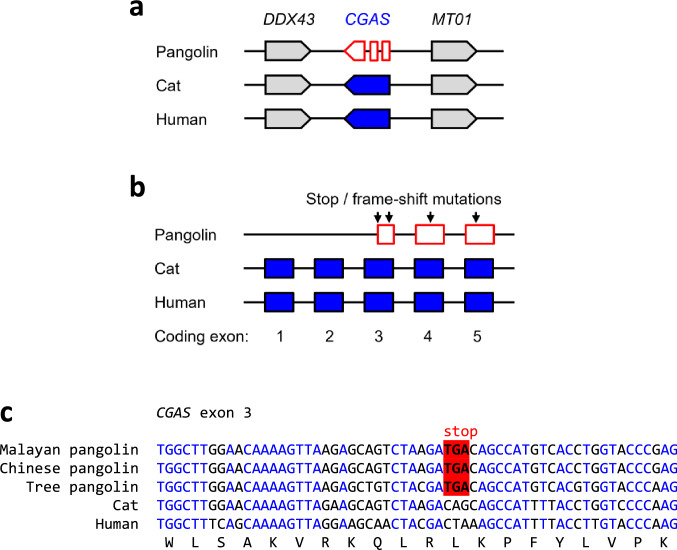
CGAS is a pseudogene in pangolins.** a** Gene locus of *CGAS* in the pangolin (*M. javanica*), cat, and human. Genes are represented by arrows pointing in the direction of transcription. A fragmented arrow indicates disruption of the coding sequence. **b** Exon-intron organization of *CGAS* in the pangolin (*M. javanica*), cat, and human. Exons containing coding sequence are indicated by boxes. Note that homologs of the first two exons could not be identified in the pangolin. Mutations leading to premature in-frame stop codons or reading frame-shifts in the pangolin gene are indicated. **c** Nucleotide sequence alignment of homologous segments of exon 3 of *CGAS* of three species of pangolins, cat and human. The coding sequence of human *CGAS* was translated and the amino acid sequence is shown below the nucleotide sequences. In-frame stop codons are highlighted by red shading. Nucleotides conserved in all species are indicated by blue fonts. Nucleotide sequence accession numbers (GenBank): Human (NC_000006.12, nucl. 73440237-73440296, compl.), cat (NC_018727.3, nucl. 69116688-69116747, compl.), Malayan pangolin (*Manis javanica*) (NW_016557438.1, nucl. 39779-39838, compl.), Chinese pangolin (*Manis pentadactyla*) (JPTV01097288.1, nucl. 22408-22467, compl.), tree pangolin (*Manis tricuspis*) (SOZM010013487.1, nucl. 43424-43483, compl.). Abbreviations: compl., complementary; nucl., nucleotide numbers (Color figure online)

Comparative genomics of mammals showed conservation of the *CGAS* gene locus in species from all major clades investigated except for the Malayan pangolin (Fig. 1a; Suppl. Table S1). The genes *DDX43* and *MT01* which flank *CGAS* in mammalian genomes, are conserved in the pangolin but no gene is annotated between them. A targeted search for *CGAS*-like sequences revealed that remnants of 3 exons of *CGAS* are located between *DDX43* and *MT01* in the pangolin (Fig. 1b). The coding sequence in each of these exon remnants was disrupted by premature in-frame stop codons and frame-shift mutations that prevent the translation into a functional protein. Analysis of the as-yet-unassembled whole genome shotgun sequences of two further pangolin species, i.e. Chinese pangolin and tree pangolin, showed also presence of disruptive mutations in *CGAS*. At least one these mutations, a premature stop codon in *CGAS* exon 3 (Fig. 1c) was conserved in all three species of pangolins, suggesting that this mutation was inherited from a common ancestor.

### STING is inactivated by gene mutations in pangolins

**Fig. 2:**
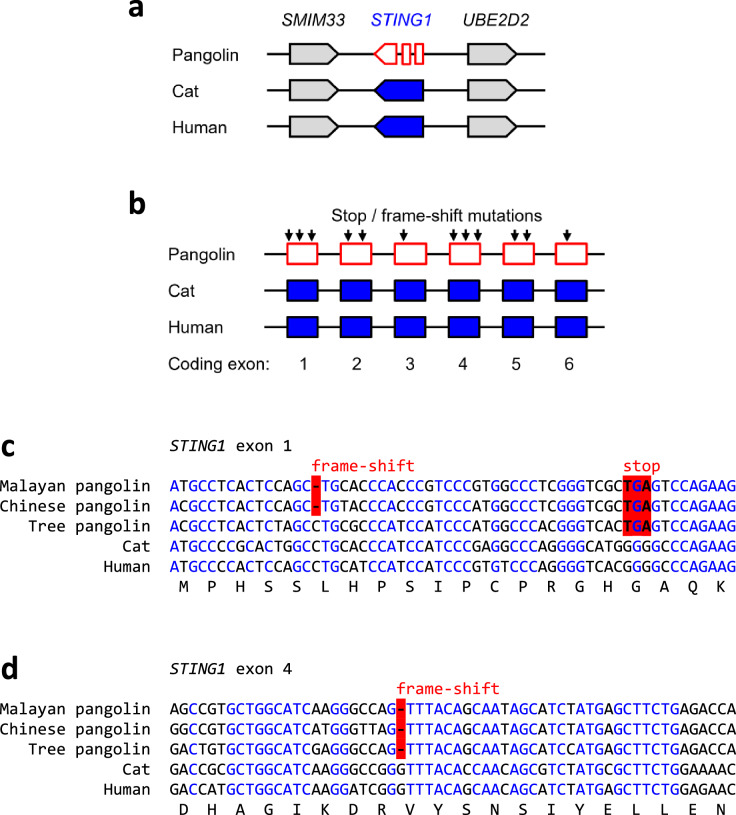
*STING1* is a pseudogene in pangolins. **a** Gene locus of *STING1* in the pangolin (*M. javanica*), cat, and human. Genes are represented by arrows pointing in the direction of transcription. A fragmented arrow indicates disruption of the coding sequence. **b** Exon-intron organization of *STING1* in the pangolin (*M. javanica*), cat, and human. Exons containing coding sequence are indicated by boxes. Mutations leading to premature in-frame stop codons or reading frame-shifts in the pangolin gene are indicated. **c, d** Nucleotide sequence alignment of homologous segments of exon 1 **(c)** and exon 4 **(d)** of *STING1* of three species of pangolins, cat and human. The coding sequence of human *STING1* was translated and the amino acid sequence is shown below the nucleotide sequences. Frame-shift mutations and in-frame stop codons in the pangolin genes are highlighted by red shading. Nucleotides conserved in all species are indicated by blue fonts. Nucleotide sequence accession numbers (GenBank): Human (NC_000005.10, nucl. 139481645-139481704, compl., 139478279-139478338, compl.), cat (NC_018723.3, nucl. 117883758-117883817, compl., 117881535-117881594, compl.), Malayan pangolin (*Manis javanica*) (NW_016540155.1, nucl. 45182-45240, 47071-47129), Chinese pangolin (*Manis pentadactyla*) (JPTV01042841.1, nucl. 8735-8793, compl., 6847-6905, compl.), tree pangolin (*Manis tricuspis*) (SOZM010002209.1, nucl. 91932-91991, compl., 90035-90093, compl.). Abbreviations: compl., complementary; nucl., nucleotide numbers (Color figure online)

Comparative genomics of mammals showed conservation of the *STING1* (the gene encoding STING) locus in all species investigated including the Malayan pangolin (Fig. 2a). However, the exons of pangolin *STING1* contained premature in-frame stop codons and frame-shift mutations that disrupted the coding sequence at multiple sites (Fig. 2b–d). Analysis of whole genome shotgun sequences of Chinese and tree pangolins showed conserved disruptive mutations in exons 1 (Fig. 2c) and 4 (Fig. 2d) of *STING1*, besides further mutations that were present in either 1 or 2 of the 3 species of pangolins investigated.

In contrast to *CGAS* and *STING1*, *AIM2* is intact in the Malayan pangolin (Suppl. Table S1) but mutated in various mammals [18–20]. IFI16, which belongs to the same protein family as AIM2 but reportedly controls DNA sensing through interactions with STING [30–32], was identified at the gene level in the cat and dog (Suppl. Table S1) but not in the Malayan pangolin (Suppl. Table S1). Genes encoding the endosomal DNA sensor TLR9 and genes controlling signaling downstream of DNA sensors such as *TBK1*, *MYD88*, *ASC*/*PYCARD*, and *CASP1* are intact in the Malayan pangolin (*M. javanica*) (Suppl. Table S1).

### The evolutionary loss of cGAS and STING occurred after the divergence of pangolins from carnivorans whereas the latter lost AIM2

**Fig. 3:**
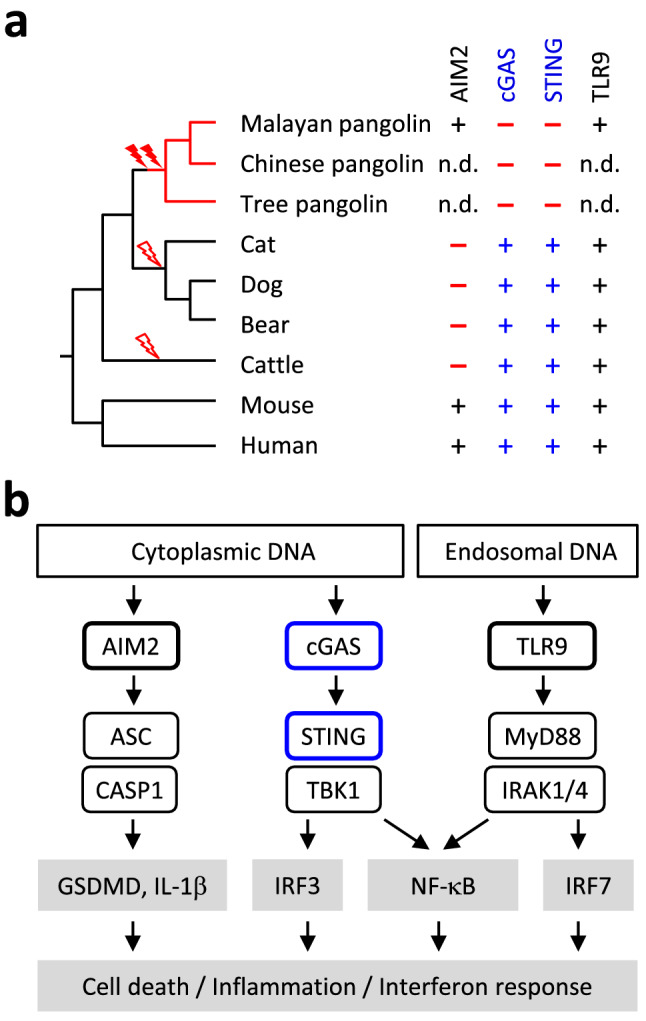
Evolution of cytoplasmic DNA signaling in pangolins and other mammals. **a** Phylogenetic tree of mammals and comparison of presence (+) or absence (–) of DNA sensor genes. Evolutionary gene loss (indicated by lightning bolt symbols) was inferred from the species distribution of the genes. Species: Malayan pangolin (*Manis javanica*), Chinese pangolin (*Manis pentadactyla*), tree pangolin (*Manis tricuspis*), cat (*Felis catus*), dog (*Canis lupus familiaris*), bear (*Ursus arctos horribilis*), cattle (*Bos taurus*), mouse (*Mus musculus*), human (*Homo sapiens*). **b** Schematic overview of innate immune sensors of cytoplasmic and endosomal DNA and signaling in mammals. Only DNA sensors investigated in this study and a subset of the signaling proteins are shown. Abbreviations: AIM2, absent in melanoma 2; ASC, Apoptosis-associated speck like protein containing a caspase recruitment domain; CASP1, caspase-1; CGAS, cyclic GMP-AMP synthase; GSDMD, gasdermin D; IL-1β, interleukin-1β; IRAK, interleukin-1 receptor-associated kinase; IRF, interferon regulatory factors; NF-κB, nuclear factor kappa-light-chain-enhancer of activated B cells; MYD88, myeloid differentiation primary response 88; STING1, stimulator of interferon genes; TBK1, TANK-binding kinase 1; TLR9, toll-like receptor 9; n.d., not determined

The next relatives to pangolins are the carnivorans, including cat, dog, bears, pinnipeds and others. All carnivorans investigated have intact *CGAS* and *STING1* genes (Suppl. Table S1) but no functional *AIM2* gene (Fig. 3). The species distribution of intact genes suggested that *CGAS* and *STING1* were inactivated by mutations in the lineage leading to pangolins and *AIM2* was lost early in the evolution of carnivorans as well as in the evolution of other mammals such as cattle (Fig. 3a). Thus, each of the two main pathways of cytoplasmic DNA sensing is inactivated in at least one clade of mammals.

## Discussion

To the best of our knowledge, pangolins are the first mammals that are reported to lack the cGAS-STING pathway. Strikingly, both genes that are specifically active in this pathway, i.e. *CGAS* and *STING1*, are pseudogenized in pangolins and the results of our comparative analysis of phylogenetically diverse pangolin species suggest that the inactivating mutations occurred more than 20 million years ago. We can conclude that the cGAS-STING pathway has been dispensable for the survival of pangolins and it is even conceivable that the loss of the cGAS-STING-dependent response to cytoplasmic DNA provided an evolutionary advantage.

We have recently reported the loss of the IFIH1/MDA5-dependent response to double-stranded RNA in pangolins [24], leading us to put forward the hypothesis that a diminished innate immune response to certain RNA viruses, including coronaviruses, may avoid overshooting host defense reactions and loss of *IFIH1* may have allowed for the evolution of tolerance as a strategy to survive viral infections. The results of the present study show that also a major pathway of sensing DNA viruses, such as herpesviruses, is inactivated in pangolins. Notably, a recent viral metagenomics study demonstrated that, besides RNA viruses, herpesviruses were abundant in pangolins [33]. Given that cGAS is the main cytoplasmic DNA sensor in non-myeloid cells, whereas AIM2 is predominantly expressed in myeloid cells [5], it is possible that epithelial target cells of viruses are particularly compromised in their antiviral response in pangolins. cGAS-STING and IFIH1/MDA5-dependent signaling pathways converge in the production of interferons, and both pathways as well as an interferon of epithelia, IFN-ε [26, 34] are lost in pangolins. Thus, an interdependent evolutionary degeneration of these pathways in pangolins is conceivable.

Importantly, cGAS and STING are not only active in antiviral defense but also in the response to mislocalization of DNA during organelle damage or defective mitosis [3, 5]. cGAS induces phosphorylation of IRF3 and stimulates apoptosis when mitosis is aberrantly arrested [3]. STING is implicated in the control of apoptosis and other modes of cell death in diverse scenarios of cell damage [5, 6]. Therefore, the absence of cGAS and STING suggests that, besides antiviral defense, the induction of programmed cell death in response to endogenous DNA is reduced in pangolins. It is also interesting to note that ZBP1, which is implicated in the response to endogenous nucleic acids [35–37], is also inactive in pangolins [24].

In contrast to the cGAS and STING pathway, the AIM2-dependent response to cytoplasmic DNA appears to be intact in pangolins. Previous studies have shown independent inactivation of AIM2 in several lineages of mammals, including the sister group of pangolins, i.e. carnivorans [18–20]. Other studies from our lab showed peculiar changes in the structure of pro-inflammatory caspases in the cat and the dog [38, 39], indicating that several components of the AIM2 pathway are altered in carnivorans. Finally, it is important to note that AIM2 is not functionally equivalent to cGAS (Fig. 3b), suggesting that loss of one of the two pathway cannot be fully compensated by the evolutionary retention of the other.

Taken together, the detection of inactivating mutations in multiple DNA or RNA-sensing proteins indicates a broad, though not complete, degeneration of the response to aberrant localization of exogenous and endogenous nucleic acids in pangolins. The role of pangolins as reservoirs of pathogens with the potential to cause zoonotic spillover and their possible use in comparative studies of immune defense and programmed cell death represent highly interesting fields for future research.

## Electronic Supplementary Material

Below is the link to the electronic supplementary material.

Supplementary Material 1 (PDF 428 kb)

## Data Availability

All data and material of this study are publicly available.
