# Supplementary material for: Cytosolic DNA sensing through cGAS and STING is inactivated by gene mutations in pangolins
Source: Apoptosis. 2020 Jun 12;25(7):474–80. doi: 10.1007/s10495-020-01614-4 (PMC7291609; doi:10.1007/s10495-020-01614-4)
Supplement: Supplementary file 1 — Supplementary Material 1 (PDF 428 kb) [file 10495_2020_1614_MOESM1_ESM.pdf]

Supplementary Table S1. Genes involved in DNA sensing and response in different mammalian species*

Species (common name)	Species (scientific name)	Gene	GenBank Gene ID	Integrity of the coding sequence
Malayan pangolin	Manis javanica	CGAS	n.a.	pseudogene
Malayan pangolin	Manis javanica	STING1	108405108	pseudogene
Malayan pangolin	Manis javanica	IFI16	n.a.	absent
Malayan pangolin	Manis javanica	AIM2	108394537	intact
Malayan pangolin	Manis javanica	TLR9	108404433	intact
Malayan pangolin	Manis javanica	CASP1	108385984	intact, protein: XP_017499094.1
Malayan pangolin	Manis javanica	DDX43	108385904	intact
Malayan pangolin	Manis javanica	MT01	108385903	intact
Malayan pangolin	Manis javanica	MYD88	108401029	intact
Malayan pangolin	Manis javanica	PYCARD	108400691	intact
Malayan pangolin	Manis javanica	TBK1	108399262	intact
Cat	Felis catus	CGAS	101098056	intact
Cat	Felis catus	STING1	101090737	intact
Cat	Felis catus	IFI16	101095311	intact
Cat	Felis catus	AIM2	n.a.	absent
Cat	Felis catus	TLR9	493839	intact
Dog	Canis lupus familiaris	CGAS	611033	intact (alternative name: MB21D1)
Dog	Canis lupus familiaris	STING1	606815	intact
Dog	Canis lupus familiaris	AIM2	n.a.	absent
Dog	Canis lupus familiaris	IFI16	488622	intact (interferon-activatable protein 203)
Dog	Canis lupus familiaris	TLR9	403502	intact
Bear	Ursus arctos horribilis	CGAS	113261117	intact
Bear	Ursus arctos horribilis	STING1	113261804	intact
Bear	Ursus arctos horribilis	IFI16	113246676	intact, protein: XP_026343122.1
Bear	Ursus arctos horribilis	AIM2	n.a.	absent
Bear	Ursus arctos horribilis	TLR9	113256623	intact
Cattle	Bos taurus	CGAS	782472	intact
Cattle	Bos taurus	STING1	533661	intact
Cattle	Bos taurus	IFI16	506759	intact
Cattle	Bos taurus	AIM2	104971426	pseudogene
Cattle	Bos taurus	TLR9	282602	intact
Mouse	Mus musculus	CGAS	214763	intact
Mouse	Mus musculus	STING1	72512	intact
Mouse	Mus musculus	IFI16	102639543	intact (ifi206)
Mouse	Mus musculus	AIM2	383619	intact
Mouse	Mus musculus	TLR9	81897	intact
Human	Homo sapiens	CGAS	115004	intact
Human	Homo sapiens	STING1	340061	intact
Human	Homo sapiens	IFI16	3428	intact
Human	Homo sapiens	AIM2	9447	intact
Human	Homo sapiens	TLR9	54106	intact

* Note: Genes mentioned in the text and in figure 3 are included.
